# Extragonadal Nongestational Choriocarcinoma With a Widespread Metastasis in a Young Female: A Case Report and Literature Analysis With a Focus on Unmet Needs

**DOI:** 10.7759/cureus.48441

**Published:** 2023-11-07

**Authors:** Amitabh Kumar Upadhyay, Shashank Shekhar, Reetal Singh, Abhishek Kumar, Vanita Pandey, Aaditya Prakash

**Affiliations:** 1 Medical Oncology, Tata Main Hospital, Jamshedpur, IND; 2 Medical Oncology, Meherbai Tata Memorial Hospital, Jamshedpur, IND; 3 Nuclear Medicine, Tata Main Hospital, Jamshedpur, IND; 4 Pathology, Meherbai Tata Memorial Hospital, Jamshedpur, IND; 5 Radiation Oncology, Tata Main Hospital, Jamshedpur, IND

**Keywords:** beta-hcg, unmet need, choriocarcinoma, extragonadal, nongestational

## Abstract

Choriocarcinoma is a highly aggressive malignant tumor that occurs due to the formation of an abnormal trophoblast. Choriocarcinoma is classified into gestational (GC) and nongestational (NGC) subtypes. The majority of nongestational diseases are limited to ovaries. Extragonadal NGC is a sporadic occurrence and a diagnostic and therapeutic dilemma. Here, we present a young 24-year-old female who presented with a widespread metastatic disease to the brain, bilateral kidneys, lungs, liver, pancreas, and small bowel. She was diagnosed with extragonadal NGC, probably originating from her kidneys. She responded poorly to standard first-, second-, and third-line chemotherapies. Detailed literature analysis with various aspects of pathogenesis, diagnostic criteria, clinical presentation, and treatment options are discussed. There is an unmet need for further research and consensus on many aspects of this rare disease.

## Introduction

Choriocarcinoma is a highly aggressive malignant tumor that occurs due to the formation of an abnormal trophoblast. It consists of two types of cells: cytotrophoblasts and syncytiotrophoblasts. The cytotrophoblasts are primitive mononuclear trophoblastic stem cells. The syncytiotrophoblasts are multinucleated cells formed by the fusion of underlying cytotrophoblasts and are the differentiated hormone-secreting components that secrete beta human chorionic gonadotropin hormone (beta-hCG) [1]. Choriocarcinoma is distinguishable as gestational choriocarcinoma (GC) and nongestational choriocarcinoma (NGC), in which the former is much more common.

GC is a rare pregnancy complication with an incidence of one in 20,000 to one in 25,000 in Western countries. Most cases of GC are intrauterine. Extrauterine GCs may also originate at a site of ectopic pregnancy in the ovary, fallopian tube, abdominopelvic cavity, or other sites. The estimated incidence of gestational ovarian choriocarcinoma is one in 369 million pregnancies. The gestational type is usually preceded by a molar pregnancy, term pregnancy, ectopic pregnancy, or abortion within one year of the antecedent event [1].

NGCs are sporadic neoplasms amounting to less than 1% of all choriocarcinomas. NGCs develop from pluripotent germ cells, most commonly in the gonads. Gonadal NGC amounts to 0.6% or less of all ovarian tumors, making it one of the rarest ovarian tumors [2,3]. Ovarian NGC is often a mixed type associated with other germ cell tumors [2,3]. Most of the NGCs in females are reported to be of ovarian or gonadal origin. Ovarian NGC is said to be common in the first to second decades of life but can be seen in any age group [2,3]. More often, the nongestational type has been seen in people who were sexually inactive, sterile, and sexually premature [2,3]. Therefore, the clinical correlation accounts for an essential part of diagnosis and treatment. 

Extragonadal NGCs are even more uncommon; are located in midline organs, such as the mediastinum, retroperitoneum, and pineal gland; and are called germ cell tumors. These are primarily reported in males and are usually associated with poorly differentiated somatic carcinomatous components [4]. Extragonadal non-midline NGCs are mainly reported from the lung, cervix, endometrium, breast, bladder, vulva, kidney, liver, pituitary, and gastrointestinal tract [2-5]. There are various hypotheses for the origin of nongonadal NGCs. These are persistent primordial germ cells with abnormal migration during embryonic development; metastasis of gonadal primary, where the gonadal primary has spontaneously regressed; origin from a trophoblastic embolus during gestation after a prolonged latency; and transformation from the primary nontrophoblastic tumor into choriocarcinoma [2-5].

The microscopic appearance of GC and NGC are similar, leading to delays in the diagnosis of NGC and often underdiagnosis or misdiagnosis in clinical practice [1,2,3]. There are no immunohistochemistry (IHC) markers to differentiate between GC and NGC. Therefore, DNA analysis and cytogenetics distinguish the two entities; excluding non-maternal DNA within the tumor confirms NGC [2-5]. It is essential to differentiate between both types as their prognosis and treatment differ [2-4]. GCs are notorious for early hematological spread, with metastasis most often seen in the lungs (80%), pelvis, vagina, liver, kidneys, and spleen. NGC metastasizes predominantly via the lymphatic system [2-4]. NGC is historically less chemosensitive and has a poorer prognosis [2-5].

Here, we report a rare extragonadal widespread metastatic NGC, probably of renal origin, in a 24-year-old lady with clinical and histopathological findings refractory to standard chemotherapy.

## Case presentation

**Figure 1:**
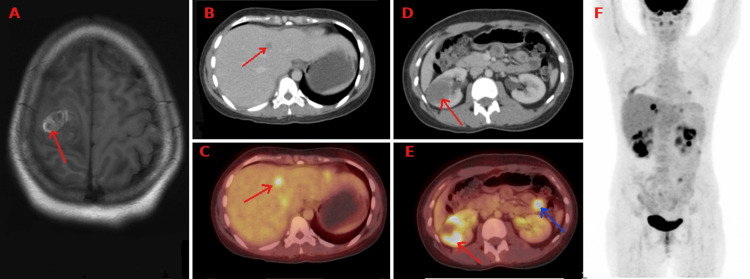
Cross-sectional MRI image (A) reveals a well-circumscribed lesion (red arrow) in the right high frontoparietal lobe within the evidence of an intralesional hemorrhagic signal. Cross-sectional CT image (B) and fused PET CT image (C) reveal FDG avid hypodense metastatic lesion in segment IV of the liver (red arrow). Cross-sectional CT image (D) and fused PET CT image (E) reveal FDG avid hypodense metastatic lesion involving the right kidney (red arrow). Images D and E also reveal FDG avid hypodense metastatic lesion involving the jejunal bowel loop (blue arrow). Maximum intensity projection (MIP) image F reveals increased foci of FDG uptake in the liver, bilateral kidneys, bowel loops, and lung regions corresponding to metastatic lesions.

A 24-year-old female, who was gravida-2, para-2, living-2, abortion-0, death-0 (G_2_P_2_L_1_A_0_D_0_) with an Eastern Cooperative Oncology Group (ECOG) performance status of one, was referred to our hospital for management of brain lesion, which was operated twice outside. Her last childbirth was 18 months before the start of symptoms, and she denied any abortion or ectopic pregnancy after her previous childbirth. She initially presented to the neurosurgery department with constant headaches, generalized seizures, and left-sided weakness. Her magnetic resonance imaging (MRI) brain was done, which was suggestive of multiple (three in number) well-circumscribed heterogeneous lesions with mildly increased diffusion signal, the intralesional subacute hemorrhagic signal in the right occipital lobe, high frontoparietal lobe, and left parietal lobe. A significant edema surrounded the lesions with a midline shift of 5 mm to the left side. The most significant lesion was 25 x 21 mm, likely neoplastic lesions (Figure 1A).

The patient underwent craniotomy under general anesthesia, and the large right parietal mass was micro-surgically decompressed. Post-craniotomy contrast-enhanced computed tomography (CECT) brain was suggestive of no midline shift, and the lesion in the right high frontoparietal lobe was not visualized as post-operative changes replaced it. Histopathology was compatible with metastatic, poorly differentiated carcinoma. After a month, she had another seizure episode and was reviewed by the neurosurgery team. Her repeat MRI brain revealed additional well-circumscribed heterogeneous lesions with mildly increased diffusion signal intralesional subacute hemorrhagic signal in the right occipital lobe and left parietal lobe. The most significant lesion measured 48 x 35 mm and was associated with edema and a midline shift of 20 mm, noted to the right side. She underwent left occipital and right occipital craniotomy and micro-surgical tumor decompression.

The patient was referred to our center for further management. Whole-body positron emission tomography and computed tomography (PET-CT) were suggestive of hypermetabolic nodules in the right lung lower lobe with other non-fluorodeoxyglucose (FDG) avid nodules likely neoplastic and minimal right pleural effusion. The right kidney showed a lesion involving the lower and interpolar cortex, approximately 4.8 x 4.5 x 5.9 cm in size, with a maximum standardized uptake value (SUV-max) of 14.2. The left kidney showed a mass lesion involving the interpolar cortex, measuring approximately 2.9 x 2.6 x 3.5 cm, with an SUV-max of 9.7. There was evidence of craniotomy with an ill-defined lesion in the parietal lobe, and an uptake is seen in the jejunal bowel loops (SUV-max 9.3). FDG avid lesions, the largest of size 1.3 x 1.1 cm, were noted in segments VIII, II, and IV of the liver. There was focal uptake noted in the tail of the pancreas, with an SUV-max of 5.0 and subtle centimeter-sized hypodensity with an SUV-max of 12.1 in the jejunal loop (Figure 1B, C, D, E).

**Figure 2:**
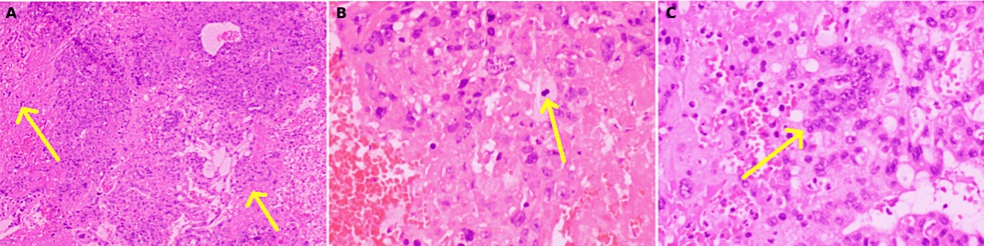
Hematoxylin & eosin (HE) staining of biopsy tissue reveals a tumor with large areas of necrosis, hemorrhage, and scanty viable components, comprising neoplastic cells in diffuse sheet-like arrangement, with focally preserved acini and cords. Cells show a marked nuclear pleomorphism, vesicular chromatin, and prominent nucleoli with conspicuous mitotic activity. Multinucleation and tumor giant cells are prominent. (A): 10x magnification (yellow arrows indicate areas of necrosis); (B): 40x magnification (yellow arrow indicates a mitotic figure); (C): 40x magnification (yellow arrow denotes the syncytial arrangement of multinucleated tumor giant cells). Overall, it is suggestive of a metastatic high-grade malignant neoplasm.

**Figure 3:**
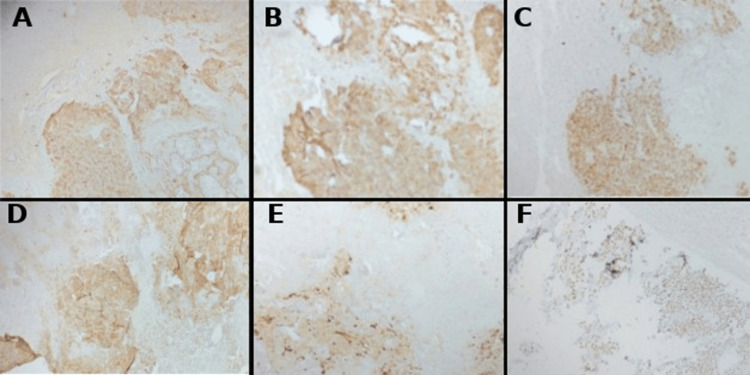
Immunohistochemistry (IHC) images showing positive stains for CK (A), CK7 (B), GATA3 (C), P63 (D), S100 (E), and SALL4 (F)

Her histopathology review of brain tumor tissue suggested a high-grade neoplasm with a differential of mixed germ cell tumor (Figure 2). Immunohistochemistry (IHC) was positive for CK, CK7, GATA3, S100, P63, and SALL-4 markers. IHC was negative for GFAP, CK20, LCA, TTF1, PAX8, CD117, AMACR, WT-1, ER, and PR, suggesting metastatic high-grade choriocarcinoma (Figure 3). Her serum beta-hCG level was 1165.5 mIU/ml. We could not do a DNA analysis of the tissue since it requires fresh tissue and resource constraints. Since there was no history of antecedent recent pregnancy, abortion, or ectopic pregnancy and the disease was outside the uterine cavity, adnexa, or ovaries, a diagnosis of widely metastatic NGC, non-gonadal choriocarcinoma was made. Bilateral kidneys were assumed to be the probable site of origin, leading to a widespread metastasis to the brain, lung, liver, and pancreas.

The case was discussed in our institutional multidisciplinary board and was planned for whole-brain radiotherapy (WBRT) and palliative chemotherapy. The patient was given WBRT in 30 Gy/10 fractions. The patient was then started on multiagent chemotherapy with a standard EMA-CO (etoposide, methotrexate, actinomycin-D, cyclophosphamide, vincristine) regime. The patient defaulted for more than two weeks after the first cycle of EMA-CO, leading to a surge in beta-hCG values to 55,027 mIU/ml before the next dose. A total of seven cycles (EMA-CO) were given with the monitoring of beta-hCG levels. The beta-hCG values never normalized, and a plateau was seen after the sixth cycle (Table 1).

**Table 1:** Serial beta-hCG values since the beginning EMA-CO: etoposide, methotrexate, actinomycin-D, cyclophosphamide, vincristine; EMA-EP: etoposide, methotrexate, actinomycin D, cisplatin; TP/TE: alternate cycles with paclitaxel, cisplatin, paclitaxel, etoposide; GEMCAP: gemcitabine plus capecitabine Table created by R Singh

Cycle number	Beta-hCG values (normal < 5.0 mIU/ml)	Regime
1	1165.5	EMA-CO
2	55027	
			14605
	2		683.01
4	599.51	
5	314.04	
6	197.91	
7	184.87	
1	721.97		EMA-EP
		632.43	
	1	72.53		TP/TE
2	4.13			
3	66.47			
4	48.84			
1	67.95	GEMCAP
2	50.41	
3	63.92	
4	80.2	
5	73.2	

**Figure 4:**
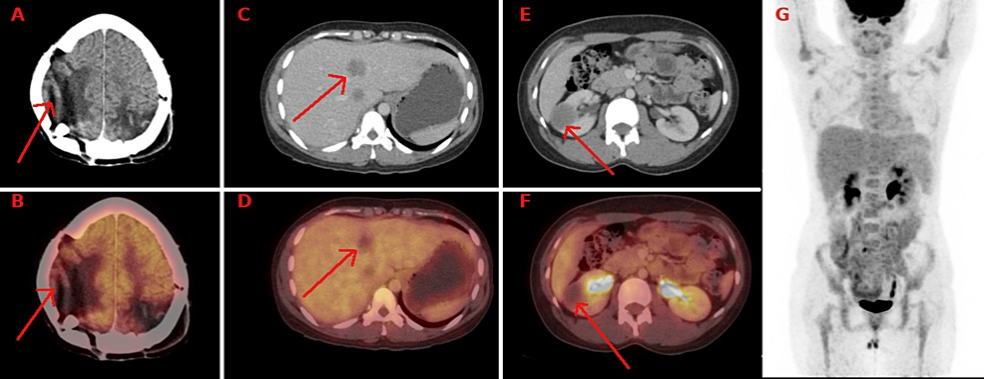
Cross-sectional CT image (A) and fused PET CT image (B) reveal postoperative changes in the right high frontoparietal lobe with no evidence of abnormal FDG avid lesion. Cross-sectional CT image (C) and fused PET CT image (D) reveal non-FDG avid hypodense lesions in the liver, suggesting healed lesions. Cross-sectional CT image (E) and fused PET CT image (F) reveal a non-FDG avid hypodense lesion involving the right kidney. Previously noted lesion involving jejunal bowel loops is not seen in the post-therapy images. Maximum intensity projection (MIP) image G reveals the resolution of previously noted foci of increased FDG uptake involving the liver, bilateral kidneys, bowel loops, and lung regions, suggesting a response to chemotherapy.

Whole-body PET-CT was done to re-assess the disease burden, showing a partial response to therapy. There was a decrease in size in most of the lung nodules and bilateral kidneys. Liver and pancreatic lesions were entirely resolved, and there was a decrease in the enhancement of previously noted brain lesions (Figure 4). Since there was a plateau in beta-hCG values, we shifted the patient to second-line chemotherapy with EMA-EP (etoposide, methotrexate, actinomycin D, cisplatin). A rising trend was seen in beta-hCG after only one cycle of EMA-EP with a value of 721 mIU/ml. She was also taking some alternative medications in between without information from our team. Since the disease was resistant to EMA-CO and EMA-EP, we started her on two weekly TP/TE regimes (alternate cycles with paclitaxel, cisplatin, paclitaxel, etoposide). She responded well to the TP/TE regime with a decline in beta-hCG levels. Beta-hCG values reduced to a nadir value of 4.1 mIU/ml in the second cycle but again increased in subsequent cycles and attained a plateau between 48 and 66 mIU/ml.

Given plateau beta-hCG levels, a response evaluation with PET-CT and MRI brain was done. PET-CT showed a stable disease, and an MRI brain suggested a post-operative cavity in the right frontoparietal lobe and the left parietal, occipital lobe, showing the nodular area of T1 hyperintensity, which shows diffusion restriction with subtle areas of enhancement.

The patient was started on a gemcitabine plus capecitabine (GEMCAP) regime, which she has received five cycles to date and tolerated well. Her recent beta-hCG level is 73.2 mIU/ml, and currently, she is asymptomatic.

## Discussion

Due to the sporadic occurrence of NGC, its clinical characteristics are unclear, and the presenting symptoms are usually nonspecific. They may present with precocious puberty in young girls. Depending upon the disease site, they can have symptoms, such as abdominal pain, abnormal uterine bleeding, pelvic lump, cough with hemoptysis, hematuria, seizures, and vision abnormalities [2-5]. Our case also presented with seizure and altered sensorium, and there was a delay in the final diagnosis.

Historically, Saito's criteria were postulated in 1963 as diagnostic criteria for NGC [6]. The four points of Saito's criteria are the absence of disease in the uterine cavity, pathological confirmation of choriocarcinoma with persistently elevated serum levels of beta-hCG, exclusion of molar pregnancy, and exclusion of coexisting intrauterine pregnancy [6]. A modified Saito's criteria was used by Shao et al. [3] in a retrospective study of 37 patients of NGC in China. This included two additional exclusion criteria. These are the history of previous ectopic pregnancies and lesions limited to the lungs with no lesions in other organs. In this study, the median age at disease onset was 22 years. Twenty-three cases denied any pregnancy history. The ovary was the most commonly affected location in 34 cases, while two cases had NGC in the pituitary, and one had NGC in the stomach. With multidisciplinary management, the overall five-year survival rate for NGC was 75.5%.

Thirty-nine cases of ovarian NGC were retrospectively analyzed by Liu et al. in 2020 [2]. The median age was 30 years, with a peak incidence in the adolescent age of 12-25 years. In this analysis, the outcome of pure NGC was better than mixed NGC in contrast to the study by Shao et al. [2,3]. Sixty-eight cases of pulmonary NGC were reported in a retrospective analysis by Cao et al. [5], which included 32 females. The clinical presentation was with cough (49.2%), dyspnea (22.2%), hemoptysis (39.7%), and chest pain (39.7%) [5]. The extragonadal NGC cases in females were also reported in the kidney, liver, urinary bladder, cervix, uterus, vulva, mediastinum, stomach, small intestines, colon, and pituitary (Table 2) [7-22]. 

**Table 2:** Published extragonadal NGC cases NGC: nongestational choriocarcinoma Table created by AK Upadhyay

Author	Country	Year	Topic/title
Yamagami et al. [7]	Japan	1983	Choriocarcinoma arising from the pituitary fossa with extracranial metastasis: a review of the literature
Matsunaga et al. [8]	Japan	1989	Primary choriocarcinoma of the stomach presenting as gastrointestinal hemorrhage: report of a case
Imai et al. [9]	USA	1994	A case of primary gastric choriocarcinoma and a review of the Japanese literature
Weiss et al. [10]	USA	2001	Primary choriocarcinoma of the vulva
Jaswal et al. [11]	India	2002	Non-gestational choriocarcinoma in small intestine
Minei et al. [12]	Japan	2008	Primary choriocarcinoma of the urinary bladder
Karadeniz et al. [13]	Turkey	2011	Bilateral renal choriocarcinoma in a postmenopausal woman
Hemati et al. [14]	Iran	2011	Choriocarcinoma of the breast; a case report and review of literatures
Malikov et al. [15]	Korea	2015	Primary hepatic choriocarcinoma in a female patient
Parker et al. [16]	USA	2016	Unusual presentation of a metastatic choriocarcinoma: a case report of non-gestational choriocarcinoma presenting as advanced colorectal cancer
Sergey et al. [17]	Bulgaria	2018	Extragonadal choriocarcinoma of the colon – Incidence, diagnosis and treatment- case report
Wang et al. [18]	China	2019	Pure nongestational uterine choriocarcinoma in postmenopausal women: a case report with literature review
Daniela et al. [19]	Brazil	2022	Difficulty in diagnosing of renal choriocarcinoma: case report
Zeinab et al. [20]	Iran	2022	Cervical choriocarcinoma in a postmenopausal woman: case report and review of literature
Orlando et al. [21]	Puerto Rico	2022	Primary non-gestational mediastinal choriocarcinoma metastatic to the brainstem
Pakkala et al. [22]	India	2023	Primary hepatic choriocarcinoma with pregnancy: a diagnostic and therapeutic challenge

Some studies propose that a prior pregnancy history should rule out a diagnosis of NGC [2-8]. A choriocarcinoma is NGC if a history of pregnancy and even previous sexual activity is ruled out. However, this is a strict criterion since most NGCs are ovarian or extragonadal germ cell tumors whose pathogenesis is unrelated to pregnancy. Hence, many patients of NGC might have a previous pregnancy unrelated to its occurrence. Recent case reports have used genetic analysis for maternal or paternal DNA to ascertain the diagnosis [23]. This can be considered a gold standard for confirmation, but the testing requires freshly processed tissue, is expensive, and is unavailable in most resource-constrained countries. Thus, the diagnosis is still based on the history and clinical presentation, which has many limitations. Many cases labeled NGC as per clinical diagnosis might have been diagnosed as GC if the genetic analysis was available and could have been done. There is a definitive unmet need for a revised criterion for differentiating GC and NGC for scientifically correct data since the therapeutic approach and outcome are very different for the two entities. 

Because of its sporadic occurrence, the staging of NGC remains a dilemma. Since ovarian or gonadal NGC is a specific type of a ovarian germ cell tumor, staging for ovarian cancer is applied in most reported cases [2,3]. There are some limitations to using ovarian staging for lung metastasis versus nonpulmonary metastatic cases. The patients with lung metastasis had better outcomes than cases with other metastasis sites [2,3]. This discrepant outcome limits the ovarian staging system for all cases of NGC. The GC staging system is another option and has been used in some case reports, but again, it has shown a poorer outcome [2,3]. For cases of NGC (ovarian and extragonadal), the WHO prognostic scoring system for malignant gestational trophoblastic diseases is irrelevant since antecedent pregnancy and interval from index pregnancy are irrelevant for these cases [1]. Thus, the literature needs more clarity regarding the ideal staging system and prognostic model for NGC cases, especially extragonadal NGC.

Some studies have shown that NGC is less chemo-sensitive and has a poorer outcome than GC [2,3,4]. EMA-CO and EMA-EP are this cohort's preferred and most commonly used chemotherapy regimes, showing good tolerance and efficacy for NGC. BEP (bleomycin, etoposide, cisplatin) and EMA-CO regimens effectively treat mixed germ cell tumors with choriocarcinoma components [2,3]. Since ovarian NGC primarily affects young females, fertility preservation should be our aim in eligible cases with unilateral disease, and the focus should be on preventing long-term complications. There are options for many second lines or subsequent line regimes, such as TP/TE, ICE (ifosfamide, carboplatin, etoposide), BEP, VIP (etoposide, ifosfamide, cisplatin), gemcitabine combinations, or immunotherapy with checkpoint inhibitors [24]. There are no separate guidelines for NGCs due to the scarcity of cases, and most of these recommendations are extrapolated from studies of GCs.

The site of origin of our case cannot be commented on with certainty, given the widespread metastasis in the brain, lungs, liver, pancreas, bilateral kidneys, and small intestine. Metastases of NGC to the kidney are rare, and bilateral metastasis is even more scarce. In our case, the most prominent lesions were noted in bilateral kidneys and can be assumed to be the primary site of origin. We could not do genetic analysis due to resource constraints for ascertaining the origin. Our diagnosis of NGC with a probable kidney origin was also based on history, histopathology, imaging, clinical presentation, and tumor behavior. The patient has responded poorly to standard regimes, such as EMA-CO, EMA-EP, TP/TE, and GEMCAP. She never had a sustained reduction of beta-hCG values, again proving the aggressive nature and poorer response of NGC compared to GC.

Next-generation sequencing (NGS) is being used for detecting various targets, which could be prognostic or predictive and may guide the development of novel therapies. Different targets, such as STK11, SMARCA4, and EGFR V774M, have been discussed by Ma et al. [25] and Onishi et al. [26]. There is further need for future research on gene mutations and signaling pathways.

There are many unmet needs or dilemmas in diagnosing and managing NGC. The preferred staging system to be applied is ovarian, or GC is unclear. No defined prognostic model exists, and the WHO GC model cannot be applied. Saito's criteria are ancient and probably need revision. The revised criteria were used by Shao et al. [3], but the diagnostic dilemma of GC versus NGC remains and probably needs further expansion/revision. The need for inclusion of genetic analysis in diagnostic criteria is imperative. How do we label the site of origin if the presentation is nongonadal and widely metastatic? Moreover, the proper selection of chemotherapy regime in the first and subsequent lines and the preferred multimodality options, especially in non-ovarian NGCs, are of utmost importance. There is a need for further research on signaling pathways and targeted therapies.

## Conclusions

Diagnosing NGC is a clinical challenge, citing a lack of proper criteria and vague clinical presentation. The role of obstetric and menstrual history is vital in diagnosis. This case highlights the vague presentation leading to delay in diagnosis and suboptimal response to standard chemotherapy options. This case has summarized the existing literature on NGC and is likely to help clinicians diagnose and manage future similar cases. Many unmet needs and dilemmas are summarized and brought to readers' attention. In the context of this case discussion, the need for further research in this area and consensus regarding diagnostic criteria and treatment options, among others, are reinforced.

## References

[1] Bishop B, Edemekong P (2023). Choriocarcinoma. StatPearls [Internet].

[2] Liu X, Zhang X, Pang Y, Ma Y, Zhang X, Liu P (2020). Clinicopathological factors and prognosis analysis of 39 cases of non-gestational ovarian choriocarcinoma. Arch Gynecol Obstet.

[3] Shao Y, Xiang Y, Jiang F (2020). Clinical features of a Chinese female nongestational choriocarcinoma cohort: a retrospective study of 37 patients. Orphanet J Rare Dis.

[4] Gao Y, Jiang J, Liu Q (2015). Extragonadal malignant germ cell tumors: a clinicopathological and immunohistochemical analysis of 48 cases at a single Chinese institution. Int J Clin Exp Pathol.

[5] Cao X, Feng H, Liu S, Chen L (2023). Analysis of clinical characteristics and prognosis of 68 patients with primary pulmonary choriocarcinoma. BMC Pulm Med.

[6] Saito M, Azuma T, Nakamura K (1963). On ectopic choriocarcinoma. World Obstet Gynecol.

[7] Yamagami T, Handa H, Takeuchi J, Niijima K, Furukawa F (1983). Choriocarcinoma arising from the pituitary fossa with extracranial metastasis: a review of the literature. Surg Neurol.

[8] Matsunaga N, Hayashi K, Futagawa S, Fukuda T, Takahara O, Yoshida K, Maeda H (1989). Primary choriocarcinoma of the stomach presenting as gastrointestinal hemorrhage: report of a case. Radiat Med.

[9] Imai Y, Kawabe T, Takahashi M (1994). A case of primary gastric choriocarcinoma and a review of the Japanese literature. J Gastroenterol.

[10] Weiss S, Amit A, Schwartz MR, Kaplan AL (2001). Primary choriocarcinoma of the vulva. Int J Gynecol Cancer.

[11] Jaswal TS, Sen R, Singh S, Punia RS, Ravi B, Arora B (2002). Non-gestational choriocarcinoma in small intestine. Indian J Gastroenterol.

[12] Minei S, Matsui T, Obinata D (2008). Primary choriocarcinoma of the urinary bladder. Hinyokika Kiyo.

[13] Karadeniz T, Topsakal M, Ozkaptan O, Cakır C (2011). Bilateral renal choriocarcinoma in a postmenopausal woman. Korean J Urol.

[14] Hemati S, Esnaashari O, Mohajeri M, Sarvizadeh M (2011). Choriocarcinoma of the breast; a case report and review of literatures. J Res Med Sci.

[15] Malikov M, Shin EA, Cho JY (2015). Primary hepatic choriocarcinoma in a female patient. Korean J Clin Oncol.

[16] Parker E, Middleton J, Garcia RL, Kilgore M, Urban R (2016). Unusual presentation of a metastatic choriocarcinoma: a case report of non-gestational choriocarcinoma presenting as advanced colorectal cancer. Ann Clin Case Rep.

[17] Iliev SD, Vladova PT, Betova TM (2018). Extragonadal choriocarcinoma of the colon incidence diagnosis and treatment - a case report. Int J Surg Med.

[18] Wang L, Wan Y, Sun Y, Zhang X, Cheng X, Wu M, Liu G (2019). Pure nongestational uterine choriocarcinoma in postmenopausal women: a case report with literature review. Cancer Biol Ther.

[19] Yela DA (2022). Difficulty in diagnosing of renal choriocarcinoma: case report [Article in Portuguese]. J Bras Patol Med Lab (Online).

[20] Nazari Z, Mortazavi L, Gordani N (2022). Cervical choriocarcinoma in a postmenopause woman: case report and review of literature. Clin J Obstet Gynecol.

[21] De Jesus O, Pellot Cestero JE, Gómez-González FM, Vélez R (2022). Primary non-gestational mediastinal choriocarcinoma metastatic to the brainstem. BMJ Case Rep.

[22] Pakkala AK, Nekarakanti PK, Nagari B, Bansal AK, Shroff G, Uppin MS (2023). Primary hepatic choriocarcinoma with pregnancy: a diagnostic and therapeutic challenge. Korean J Gastroenterol.

[23] Stockton L, Green E, Kaur B, De Winton E (2018). Non-gestational choriocarcinoma with widespread metastases presenting with type 1 respiratory failure in a 39-year-old female: case report and review of the literature. Case Rep Oncol.

[24] Alazzam M, Tidy J, Osborne R, Coleman R, Hancock BW, Lawrie TA (2016). Chemotherapy for resistant or recurrent gestational trophoblastic neoplasia. Cochrane Database Syst Rev.

[25] Ma Y, Wang C, Sun PL, Zhu Y, Huang ZK, Jin SX (2018). A case of male primary pulmonary choriocarcinoma. Chin Med J (Engl).

[26] Onishi I, Kirimura S, Wakejima R (2022). Primary pulmonary choriocarcinoma with a genomic sequence. Pathol Int.

